# An integrated care programme in London: qualitative evaluation

**DOI:** 10.1108/JICA-02-2018-0020

**Published:** 2018-10-15

**Authors:** Thomas Round, Mark Ashworth, Tessa Crilly, Ewan Ferlie, Charles Wolfe

**Affiliations:** 1 School of Population Health & Environmental Sciences, Faculty of Life Sciences & Medicine, King’s College London, London, UK; 2 Crystal Blue Consulting Ltd, London, UK; 3 King’s Business School, King’s College London, London, UK

**Keywords:** Integrated provision of care, Integrated health and social care

## Abstract

**Purpose:**

A well-funded, four-year integrated care programme was implemented in south London. The programme attempted to integrate care across primary, acute, community, mental health and social care. The purpose of this paper is to reduce hospital admissions and nursing home placements. Programme evaluation aimed to identify what worked well and what did not; lessons learnt; the value of integrated care investment.

**Design/methodology/approach:**

Qualitative data were obtained from documentary analysis, stakeholder interviews, focus groups and observational data from programme meetings. Framework analysis was applied to stakeholder interview and focus group data in order to generate themes.

**Findings:**

The integrated care project had not delivered expected radical reductions in hospital or nursing home utilisation. In response, the scheme was reformulated to focus on feasible service integration. Other benefits emerged, particularly system transformation. Nine themes emerged: shared vision/case for change; interventions; leadership; relationships; organisational structures and governance; citizens and patients; evaluation and monitoring; macro level. Each theme was interpreted in terms of “successes”, “challenges” and “lessons learnt”.

**Research limitations/implications:**

Evaluation was hampered by lack of a clear evaluation strategy from programme inception to conclusion, and of the evidence required to corroborate claims of benefit.

**Practical implications:**

Key lessons learnt included: importance of strong clinical leadership, shared ownership and inbuilt evaluation.

**Originality/value:**

Primary care was a key player in the integrated care programme. Initial resistance delayed implementation and related to concerns about vertical integration and scepticism about unrealistic goals. A focus on clinical care and shared ownership contributed to eventual system transformation.

## Introduction

An ageing population with increasing prevalence of multi-morbidity, rising costs of technology and budgetary pressures are all placing a significant strain on health care systems (NHS England, 2014). Integrated care has become a central part of health service reform in response to these challenges (Alderwick *et al.*, 2015), and is an organising principle for care delivery with the aim of achieving improved patient care through better co-ordination of services in primary care and in the community (Shaw *et al.*, 2011); however, there is no single, agreed model (Robertson, 2011). Integrated care can take many different forms, involving whole populations, care for particular groups or people with the same diseases, or co-ordination of care for individual service users and carers (Ham and Curry, 2011). It can focus on a model bringing together primary and secondary care or involving a wider alliance of health and social care, with the most complex forms bringing together responsibility for commissioning and provision (Ham and Curry, 2011).

Current evidence suggests that integrated care is of most importance for people with multi-morbidity (Wallace *et al.*, 2016), where there is a risk that care will be fragmented and deliver poor outcomes. In contrast, the benefits of large-scale integration in social health care have yet to be seen (Bardsley *et al.*, 2013). There is a lack of robust evidence both for the cost-effectiveness of integrated health care (Local Government Association, 2013), and for the integration of health and social care (National Audit Office, 2017). Previous research has measured several aspects of integrated care, including access to health care services, clinical outcomes and cost-effectiveness. However, there is a relative lack of qualitative research in this field (Mastellos *et al.*, 2014; Kassianos *et al.*, 2015).

### Rationale and context

This evaluation involved a programme launched in two adjacent inner-city London boroughs with a combined population of over 400,000. The programme faced substantial challenges including a socioeconomically deprived and multi-ethnic population, a high burden of disease and fragmented services. In response to these challenges, a “partnership programme” was formed in 2012 with the initial aim to achieve a 14 per cent reduction in emergency bed days per month together with an 18 per cent reduction of residential care home placements. This would, in turn, release large savings projected to be almost £14m per annum by year 4 (2015–2016) (Southwark and Lambeth Integrated Care (SLIC), 2016). Programme funding was approximately £27.5m over four years and was derived from three sources: the two Clinical Commissioning Groups (CCGs) were equal contributors and a local charitable trust was the largest contributor (SLIC, 2016). In spite of high initial expectations, it became clear that large-scale reductions in secondary care utilisation and residential care placements were unlikely to be achieved. In the second year of the programme, targets were “re-profiled” to be replaced by more modest overall goals for secondary and residential home care utilisation and a focus on feasible service integration.

### Structure and governance

Programme health service partners included: all local general practices, three NHS Foundation Hospital Trusts, one Mental Health Foundation Trust, two CCGs and two local council authorities.

The programme had four main boards: a Sponsor Board provided strategic direction and high-level decision making; a Provider Group dealt with how to turn strategy into action, and acted as a Programme Board; an Operations Board oversaw delivery; a Citizens’ Board provided input from patients and local citizens. Citizens were represented on each board as was primary care.

### Interventions

**Figure 1:**
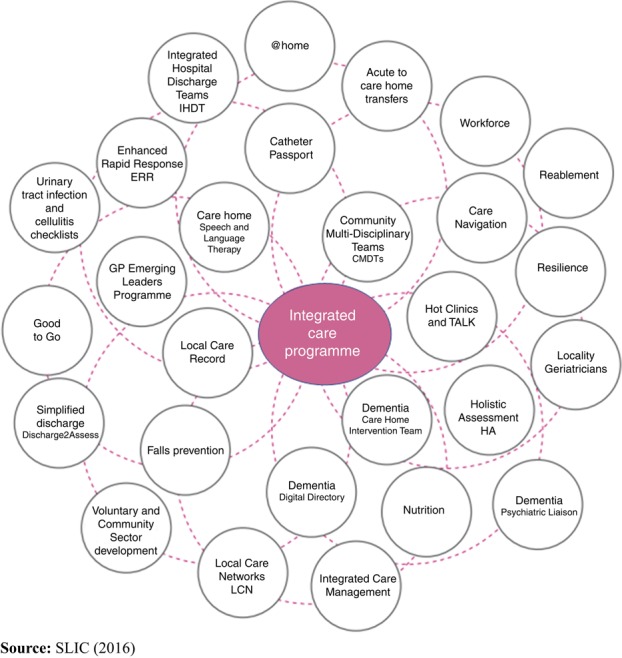
The integrated care programme

This was a complex programme consisting of multiple interventions across primary, secondary and social care. These interventions are summarised in Figure 1.

The programme interventions included:

1. Holistic health assessments in primary care focussed on the over 65-year old population; assessments consisted of a review of physical health, mental health, social care and self-care needs; a universal template was devised to capture and record the data in electronic primary care records.
2. The Local Care Record (LCR): an IT solution created to allow read-only access between primary care, secondary care and mental health case records. The LCR enabled care providers to view clinical records, correspondence, prescribing, investigation findings (e.g. blood test, radiology and histology results) and follow-up appointments.
3. Integrated care management: patient-level integrated care under the co-ordination of a community-based care manager responsible for overseeing the linkage of care across organisational boundaries for individual patients.
4. Community Multi-Disciplinary Teams: regular (usually monthly) case management meetings taking place in the community and attended by general practitioners, practice nurses, district nurses, elderly care physicians, psycho-geriatricians, social services.
5. Development of clinical pathways in the community, including for falls, infection, nutrition and dementia; designed to maximise community-based care and avoid secondary care attendance.
6. The Older Persons Programme (OPP) contained the following components of integrated care:
  - Enhanced Rapid Response teams who provided enhanced therapy, nursing and social care, to help people stay independent in their own homes.
  - “@home”, a multi-disciplinary team providing holistic, integrated care for acutely unwell patients at home who would otherwise require hospital admission.
  - Reablement, a service for all residents who qualified for a Domiciliary Care Package on discharge from hospital. It aimed to help people regain their independence. This provision was integrated with the Community Health Supported Discharge Team.

### Evaluation

The aims of the evaluation of the integrated care programme were:

1. To identify what worked well and what did not in terms of developing integrated care.
2. To determine the lessons learnt from the programme.
3. To gain an understanding of the value of investment in integrated care.

## Methods

Qualitative evaluation described in this case report was part of a wider mixed methods evaluation using quantitative and qualitative data gathering and analysis coupled with a health economic assessment (SLIC, 2016). Further details of the economic assessment are also contained in the report (SLIC, 2016). The evaluation ran from January to May 2016.

Documentary evidence was scrutinised including the original business case, interim progress reports, reports to funders, board papers and minutes of meetings and previous evaluation reports.

Qualitative data were also obtained from recorded semi-structured interviews, focus groups and stakeholder meetings. Audio files and contemporaneous notes were shared by the research team. Notes were kept of integrated care meetings attended by the evaluation team. Purposive sampling (Mays and Pope, 1995) was used to guide our approach to data gathering. Following the purposive approach, interviewees were selected on the basis of known engagement with the structures, processes and outcomes of integrated care.

The evaluation team conducted 31 semi-structured interviews including: three citizen representatives; two from central management team; two from charity partner/funder; three from local authorities; six from local secondary care providers (three providers); three hospital consultants (two providers); five general practitioners/general practitioner Federation leads; three community providers; and four commissioners/CCG representatives.

The interviews were conducted by four members of the evaluation team and ranged between 30 and 70 min in length, leading to over 25 hours of recorded interviews.

All conversations were digitally recorded with consent, with researchers taking field notes during the interviews and meetings. The consent process required that all quotes would be un-attributable/non-identifiable.

### Previous evaluation findings

Findings were reviewed from three prior commissioned evaluations and, together with observational data from stakeholder meetings, contributed to the development of the interview Topic Guide (King, 2015; Ross, 2015; RAND, 2016).

### Topic guide

For the semi-structured interviews, the following open questions were used as a guide to help direct the questions:

- Successes/Strengths/Facilitators: what have been the strengths of the programme?
- Challenges/Weaknesses/Barriers: what did not work and why?
- Lessons learnt: what would you do differently in the future?

Combining different methods of qualitative research (documentary analysis, semi-structured interviews and focus groups) allowed for further examination of patterns of convergence and corroboration (Lambert and Loiselle, 2008; Mays and Pope, 2000). Focus groups also allowed the interaction and perceptions resulting from discussion amongst participants to be examined (Pope *et al.*, 2002). Data were thematically analysed using the framework approach (Ritchie *et al.*, 1994; Srivastava and Thomson, 2009). Analysis and validation of the emergent themes were conducted by all members of the interview team, improving consistency and reliability. As the themes emerged, these were discussed and cross-checked during subsequent interviews and focus groups as a form of respondent validation to increase validity (Pope *et al.*, 2000).

## Results

### Overarching themes: “successes and challenges” and “lessons learnt”

The key themes of “successes and challenges” to the integrated care programme and “lessons learnt” are presented in Table I and each in turn is discussed in more detail.

### Theme 1: shared vision and case for change

Successes and challenges: successes included overall positive ideas about the programme, such as attempting to deal with issues around rising hospital admissions for frail older persons, and nursing home placements. One senior manager reported, “Life now looks very different; in a very positive way”. The overall investment and upscaling including of primary care was felt by stakeholders to be a real success: “scaling up of primary care makes sense, but needs adequate resource”, and “the investment has increased capacity in the system”.

Challenges included a feeling that the programme had, “massively overambitious proposals in the original business case” and was “too ambitious with a lack of realism”. This hampered progress to deliver the initial objectives: “implementation was slow in beginning, the business case was too optimistic about potential to achieve early on”. The focus of the programme was noted to have “[…] changed over time. This was to retain focus of older persons’ programme and explore long-term conditions in a general sense and resilience and wellbeing”. There was also felt to be a lack of communication between leadership of the programme and operational delivery with, “a disconnect between the Sponsor Board and the level below”, leading to it being, “harder to find the common ground”.

Lessons learnt from stakeholders included that for future integrated care programmes, it would be important to keep it simple and, “less complex and smaller scale initially”, with, “a clarity of purpose and vision”. Recommending, “a clear view of limited things and do them well”, with, “no appetite for a big central team”.

### Theme 2: interventions

A number of interventions were perceived as successes, particularly interventions from the OPP, described as “a real success”. Bridging the gap between primary and secondary care was also frequently mentioned, including, “this got geriatricians out of the ivory tower to connect with general practice”, and “the locality geriatrician meant easier access to advice for general practitioners”. Improved information technology such as the LCR was also felt to be a tangible success: “IT changes have helped and have now been rolled out across general practices”. However, implementation, “has taken quite a few years”, with benefits taking time to accrue.

There were also a number of interventions identified as challenges and barriers to rapid implementation of the programme. Holistic assessments were felt to be, “a very lengthy assessment”, and “hugely dependent on the individual doing them”, whilst, “some viewed this as tick box exercise”. There were observations that they had not been, “piloted or iteratively developed and we didn’t test design”, “[…] or test the hypothesis”. Community multi-disciplinary team meetings (between primary and secondary care) were also felt to have been the, “most important and deliverable aspect”, and an, “extreme example of something that could have been so good”, and yet had become, “an exercise in doing whatever further up felt they should”, which, “rarely did anything useful”. Long-term conditions included in the original business case did not feature strongly in the programme, and it was reported to have “missed long term conditions and complexity in younger people”.

Lessons learnt included that interventions “should be evidence based, and be explicit when generating new evidence”, with the, “need to review the change model, and almost do mini randomised controlled trials and pilot and iteratively develop these”, and to “pilot and test delivery of interventions with different staff members”. It was felt that targeting long-term conditions would be the key for the success of future integrated care programmes, and they should, “start with the health of the population, start with strong public health and primary care”.

### Theme 3: leadership

Leadership was a strong overall theme from many interviews, including both successes and challenges of the programme. Clinical leadership in primary care was considered by many to, “have been a real benefit”, including primary care engagement and leadership on the programme boards. During the course of the programme, the charity had initiated a separately funded primary care leadership programme which contributed to the professional development of those leading integrated care: “investment in primary care emerging leaders programme (was) an important catalyst”.

Some interviewees reported, “a clash between management led and clinical led models”, and questioned: “was there enough clinical leadership?” There was also a lack of communication, “between the leadership and what happened on the ground”, with, “execs working at high level strategic level, but virtually no help or guidance to those on the ground”.

Lessons learnt included that “stronger clinical leadership is needed”, and that “future proposals have to come from and be owned by primary care”. Also, the, “need for time and resource for planning”, and to be able to, “innovate and to be able to move from proactive to reactive care”.

### Theme 4: relationships

Collaborative working and culture change was perceived as a shared success, as a great strength of the programme, and of its legacy. “Relationships have been built up”, with, “[…].(the) main strength to help us build relationships between primary care, secondary care, community services and social services”. This included shared learning and “co-production between different staff and users”, which developed over time.

However, there was, “initial hostility and suspicion on both sides”, with, “primary care worried about a takeover”, reacting with, “hostility to what felt like a […] secondary care thing”, whilst “the complexities of general practice were not properly understood”. This potential for mistrust appeared to shift over time to a more collaborative working culture.

In terms of lessons learnt, stakeholders reported the need to be involved: “organisations need to have ownership”, and with a shared culture, “ownership and co-production”.

### Theme 5: organisational structures and governance

Primary care networks and federations which developed alongside the programme were reported to be a key success based on stakeholder interviews: “scaling up of primary care into networks makes sense”. Programme organisational structures were thought to be too complex with some stakeholders reporting they, “never fully understood who does what”, “it was too offshore” and “[…]. (the programme) became a bit of everything”. The governance structures were also perceived, “not to be embedded enough in organisations, and allows them to not own or not contribute”.

Lessons included criticism that the programme was too top down with the need for a “bottom up approach, not branded as different from (the) day to day job”. There were also a number of views on governance including the need to bring, “budgets together that allows accountability for each section and encourages good performance in those areas”, and to, “start with commissioners spending money for maximum benefit”, with “incentives following outcomes, not just activity”.

### Theme 6: citizens and patients

The local citizen involvement within the programme was reported as a success with the, “citizen voice present in all spheres of decision making”; “compared to most programmes the citizens were at the heart of this” and “engagement is something to be proud of”. However, some stakeholders challenged whether, “the citizens’ board was representative”, and “the role of citizens has not always been clear”. Concerns were also raised about potential conflict between the roles of citizens and service users as patients.

Lessons included the potential for conflict expressed between the agenda of citizens and service users as patients with, “the patient journey, experience and shared agenda were all lacking”, and “a clearer role for citizens needed”.

### Theme 7: evaluation and monitoring

There were several external evaluations with one interviewee commenting, “it was insisted that the evaluations were outsourced and this was not managed well”, with, “a lot of money spent on external agencies and management consultants. Was this money well spent?” Also, reported was the perception that, “no one pushed the evaluations to embed”, and “despite the knowledge available we didn’t change the focus of the programme enough”.

It was reported that similar complex integrated care programmes in the future would benefit from, “continuous academic input and evaluation”, which “need both research and implementation”. Some stakeholders mentioned the researcher in residence model as an example of how this might work in practice.

### Theme 8: macro-level environment

For many stakeholders, the macro-level environment was an important theme, particularly focussing on the, “slashing of local authority budgets”, and “cuts to primary care and mental health budgets”, which meant it was, “difficult to deliver social care integration”. With the external environment reported as making, “the system dysfunctional”, this hampered the ability of organisations to deliver innovation which spanned boundaries within the programme.

Lessons learnt included that any future integrated care programmes need to take into account the macro environment and policy context, with contingency planning. Planned local interventions and structures need to be kept simple especially with limited resources and complex macro-level changes.

## Discussion

This evaluation of a complex integrated care programme offers insights into large-scale change across multiple health and social care organisations working in the same community. The programme had to overcome inherent tensions both within and between the health and social care system which hampered shared working.

There is a choice to be made in balancing successes against challenges. Greenhalgh *et al.*’s (2009) realist evaluation of system transformation did not adjudicate between success and failure, noting that these concepts are “socially negotiated”. In a large complex system, there will be continued differences in perception and incomplete knowledge. Areas that remain contested included: the clinical evidence base underpinning the original business plan; the impact of interventions on hospital utilisation; the extent to which data are used to systematically drive improvement at the delivery locus; the provenance and origin of change and whether the inception of some interventions pre-dated the programme.

Nevertheless, there was a consensus based on interviews that the project provided a vehicle for strengthening and unifying delivery of care for older people. Funding had created a space (releasing human resource) that enabled time and effort to be invested in developing integrated care.

In balancing costs and benefits, many of which were intangible due to the nature of data collected, there were two schools of thought among stakeholders. One was that in a complex environment, investment in the programme provided an opportunity to experiment and innovate; it delivered activity; it developed relationships and connections that contributed to system transformation preparing it for the future. Most interview subjects emphasised the proposition that the system had become stronger and better placed than it would have been without this initiative.

The second and divergent opinion was that the costs did not realise the projected benefits and that the health gain related to some interventions remained uncertain. Clarity about benefit was hampered by the lack of a clear evaluation strategy and of the evidence required to corroborate claims of benefit.

It could be argued that the integrated care programme made progress in developing each of the three components reported to constitute high performing networks (Ferlie *et al.*, 2011): IT developments which began to come on stream near the end of the project; a narrative journey which demonstrated inter-organisational learning and adaptability; and the development of broader more horizontal governance structures. In spite of achievements in these components, achievement was incomplete. The IT developments only contributed to the delivery of integrated care in the final few weeks of the programme; inter-organisational conflict may have detracted from the learning opportunities; and although there was evidence of more horizontal structures, a frequent criticism was the top-heavy, top-down nature of the programme.

### Comparison with existing literature

#### Integrated care

In a review of integrated care programmes (Dorling *et al.*, 2015), four key factors for success were identified: patient education/empowerment; care co-ordination; multi-disciplinary teams; and individual care plans. Our investigation found elements of all four factors within the themes identified. A more recent review (Wallace *et al.*, 2016) of community-based interventions for reducing emergency admissions identified three potential approaches: targeting specific long-term conditions, end of life care and early discharge schemes. Based on our data, the programme only focussed on one of these approaches, the development of early discharge schemes.

It has been argued that integration should start with a focus on service users at the centre, rather than from organisational solutions (Ham and Oldham, 2009) and that, “the patient’s perspective is at the heart of any discussion about integrated care (and) […] the organising principle of service delivery” (Shaw *et al.*, 2011). A conceptual framework for understanding integrated care has been proposed which places person-focused care with clinical integration at the centre of the process (Valentijn *et al.*, 2013). Although the programme included substantial citizen engagement within its organisational structure, it would be difficult to conclude, based on our findings, that the patient perspective had become “the organising principle” shaping the development and implementation of integrated care.

### Evaluation and monitoring

Themes from the interview evidence included proposals that future schemes should incorporate inbuilt evaluation. This approach could iteratively adapt and develop the evidence base, and feed into the development process, such as the “researcher in residence” model (Marshall *et al.*, 2014). It has been argued that established approaches to translating health services research evidence into practice have not significantly influenced management decisions (Rycroft-Malone *et al.*, 2011) highlighting the need for further evidence-based policy and implementation (Black and Donald, 2001) to drive quality improvement. A common feature of proposed researcher in residence models is the concept of “co-creating” knowledge between researchers, practitioners and managers.

An important aspect of the design of an evaluation is the choice of outcome measures, linked to a theoretical understanding of the intervention. In the case of this programme, it could be argued that the primary measures were around process and activity rather than patient-centred care, and that in retrospect they were over-optimistic and unlikely to be achievable.

### Structures and ownership

From the interview evidence, the structure of the programme was criticised as too complex and top down. This recognition led to continued effort to engage general practitioners. Similarly, Ham and Walsh (2013) concluded that integrated care should be built, “from the bottom up as well as the top down”, with the main benefits occurring when barriers between services and clinicians are broken down, not when organisations are merged. The interview evidence suggested initial mistrust from primary care particularly related to concerns about “vertical integration”. By the time the project concluded, these concerns had largely been addressed.

Another emergent theme was the need for shared ownership and leadership, which again is consistent with the evidence about integrated care where, “whole-system working needs to be based on sound governance arrangements with clarity around decision making and accountability” (Ham and Walsh, 2013).

A success identified from stakeholder interviews was the development of primary care networks/federations. There is developing evidence that the move towards primary care federations may facilitate integration (Addicott and Ham, 2014) and lead to better outcomes for long-term conditions (Hull *et al.*, 2014; Pawa *et al.*, 2017). Again, causal relationships are difficult to attribute as the formation of networks/federations overlapped with the programme interventions. It is likely that configurations of networks/federations would have developed without the impetus of delivering an integrated care programme.

### Looking to the future: recommendations

Based on the findings of our evaluation, we make the following seven recommendations:

1. The strength of the evidence base should be made explicit. Strong evidence should be grounded in the literature that specifies how the intervention might improve care and outcomes. Innovation that does not yet have a published evidence base would benefit by being clearly badged as such.
2. The rationale for interventions should be linked to population need.
3. Quality improvement methods should be built into new programmes.
4. Outcome measures should be defined in agreement with stakeholders.
5. Evaluation should be designed at the planning stage, setting up a relationship between planned intervention and proposed outcomes enabling costs and value to be assessed.
6. The role of citizen involvement should be clarified, with consideration of how to maximise representativeness of the population.
7. The proposed definition and measurement of value should be articulated.

## Conclusions

This qualitative evaluation has demonstrated both the successes and challenges of an ambitious four-year integrated care programme.

Our evaluation was conducted against the background of growing acceptance that this generously funded integrated care programme was not going to deliver the expected radical reductions in secondary care and nursing home utilisation (16). Nevertheless, most partners within the programme emphasised the proposition that system transformation has been achieved in terms of shared working between health and social care organisations and whole-system working.

Evaluation is a key component of organisational change, including integrated care. Our retrospective evaluation has emphasised the importance of prospective evaluation from conception to conclusion, more akin to “quality improvement” models.

The lessons learnt have important implications for others seeking to embark upon integrated care initiatives of equivalent scale. Indeed, various English large-scale integrated care schemes are at differing stages of completion and an overview of their findings would be a helpful next step in building a more complete knowledge base.

## Figures and Tables

**Table I:** Summary of overarching themes: successes and challenges of the programme; lessons learnt

Theme	Successes	Challenges	Lessons learnt
Shared vision/case for change	Positive ideas and direction Primary care investment and upscaling	Business case overambitious Change and lack of focus and ownership Disconnect between leadership and operational delivery	Keep it simple, smaller scale, focused
Interventions	OPP interventions including Enhanced Rapid Response teams, Reablement, “@home” service Community Geriatricians, Catheter care/passport, Community Multi-Disciplinary Teams Stabilising of admission rates Information Technology/Local Care Record	Holistic assessment — not targeted Overly process driven Community Multidisciplinary Teams were difficult to develop Long-term conditions not included IT slow to accrue benefits	Evidence based (adapt and generate), co-designed, piloted with iterative development. Focused interventions
Leadership	Clinical leadership: primary care	Management vs clinical leadership Disconnect between strategic leadership and operational delivery	Clinical leadership Time and resources
Relationships	Collaborative working and culture change	Mistrust — resistance particularly amongst primary care	Stakeholder involvement and ownership Shared culture
Organisational structures and governance	Primary care networks/Federations	Complexity, top down	Bottom up (and top down) Ownership Budgetary incentives
Citizens and patients	Citizen involvement	Representativeness	Patients as partners Service users and carers Self-management
Evaluation and monitoring	Shaped the process	Multiple external evaluations, opportunity costs	Inbuilt evaluation, researcher in residence Review and develop the evidence base
Macro level	Integrated care a national priority. Ageing population. Vanguards, publication of *Five Year Forward View*	Austerity/Local Authority cuts Health and Social Care Act	Context and contingency planning Keeping interventions and structures simple
